# Supplementary material for: Validation of global precipitation time series products against tree ring records and remotely sensed vegetation greenness
Source: PLoS One. 2024 Feb 29;19(2):e0299111. doi: 10.1371/journal.pone.0299111 (PMC10903888; doi:10.1371/journal.pone.0299111)
Supplement: Attachment — Submitted filename: 2023-11-09 Respose to reviewers.docx [file pone.0299111.s001.docx]

Dear Dr. Manvailer,

Thank you for submitting your manuscript to PLOS ONE. After careful consideration, we feel that it has merit but does not fully meet PLOS ONE’s publication criteria as it currently stands. Therefore, we invite you to submit a revised version of the manuscript that addresses the points raised during the review process.

Please submit your revised manuscript by Jul 20 2023 11:59PM. If you will need more time than this to complete your revisions, please reply to this message or contact the journal office at plosone@plos.org. When you're ready to submit your revision, log on to https://www.editorialmanager.com/pone/ and select the 'Submissions Needing Revision' folder to locate your manuscript file.

We look forward to receiving your revised manuscript.

Kind regards,

Nir Y. Krakauer

Academic Editor

PLOS ONE

Our apologies that this has taken a little longer than expected. One suggestion made by both reviewers was to include another high-quality remote-sensing based product, as the PERSIANN dataset was not representative of the current state of the art for precipitation estimations. This required a complete re-run of the remote sensing-based evaluation. We are glad that we expanded our analysis, however. The recommended dataset (MSWEP) performed very well, with improvements especially pronounced where weather station coverage is generally low (e.g. Africa).

**Journal Requirements:**

When submitting your revision, we need you to address these additional requirements.

1. Please ensure that your manuscript meets PLOS ONE's style requirements, including those for file naming. The PLOS ONE style templates can be found at

https://journals.plos.org/plosone/s/file?id=wjVg/PLOSOne_formatting_sample_main_body.pdf and

https://journals.plos.org/plosone/s/file?id=ba62/PLOSOne_formatting_sample_title_authors_affiliations.pdf

We read the requirements and made adjustments where applicable

2. Please note that PLOS ONE has specific guidelines on code sharing for submissions in which author-generated code underpins the findings in the manuscript. In these cases, all author-generated code must be made available without restrictions upon publication of the work. Please review our guidelines at https://journals.plos.org/plosone/s/materials-and-software-sharing#loc-sharing-code and ensure that your code is shared in a way that follows best practice and facilitates reproducibility and reuse.

We note that all data we use in this study are already open access. That said, we also deposited our code and intermediate data products (e.g. extracted and summarized remote sensing data) in an open access repository. The link has been included in the manuscript and in the cover letter: <https://doi.org/10.6084/m9.figshare.24540928>.

3. Thank you for stating the following in the Acknowledgments Section of your manuscript:

"We thank the custodians and all contributing authors of the International Tree Ring Database for creating this valuable resource. Funding for this study was provided by the NSERC Discovery Grant RGPIN-330527-20 to AH and a Coordenadoria de Aperfeiçoamento de Pessoal de Ensino Superior (CAPES) scholarship to VM."

We note that you have provided funding information that is not currently declared in your Funding Statement. However, funding information should not appear in the Acknowledgments section or other areas of your manuscript. We will only publish funding information present in the Funding Statement section of the online submission form.

Please remove any funding-related text from the manuscript and let us know how you would like to update your Funding Statement. Currently, your Funding Statement reads as follows:

"A.H. RGPIN-330527-20 Natural Sciences and Engineering Research Council of Canada (NSERC)

https://www.nserc-crsng.gc.ca

The funders had no role in study design, data collection and analysis, decision to publish, or preparation of the manuscript."

Please include your amended statements within your cover letter; we will change the online submission form on your behalf.

We deleted that sentence in the acknowledgement section.

4. In your Data Availability statement, you have not specified where the minimal data set underlying the results described in your manuscript can be found. PLOS defines a study's minimal data set as the underlying data used to reach the conclusions drawn in the manuscript and any additional data required to replicate the reported study findings in their entirety. All PLOS journals require that the minimal data set be made fully available. For more information about our data policy, please see http://journals.plos.org/plosone/s/data-availability.

"Upon re-submitting your revised manuscript, please upload your study’s minimal underlying data set as either Supporting Information files or to a stable, public repository and include the relevant URLs, DOIs, or accession numbers within your revised cover letter. For a list of acceptable repositories, please see http://journals.plos.org/plosone/s/data-availability#loc-recommended-repositories. Any potentially identifying patient information must be fully anonymized.

Important: If there are ethical or legal restrictions to sharing your data publicly, please explain these restrictions in detail. Please see our guidelines for more information on what we consider unacceptable restrictions to publicly sharing data: http://journals.plos.org/plosone/s/data-availability#loc-unacceptable-data-access-restrictions. Note that it is not acceptable for the authors to be the sole named individuals responsible for ensuring data access.

We will update your Data Availability statement to reflect the information you provide in your cover letter.

We note that all data we use in this study are already open access. That said, we have now deposited our code and intermediate data products (e.g. extracted and summarized remote sensing data) in an open access repository. The link has been included in the manuscript and in the cover letter: <https://doi.org/10.6084/m9.figshare.24540928>.

5. We note that Figures 1,2 and 4 in your submission contain map/satellite images which may be copyrighted. All PLOS content is published under the Creative Commons Attribution License (CC BY 4.0), which means that the manuscript, images, and Supporting Information files will be freely available online, and any third party is permitted to access, download, copy, distribute, and use these materials in any way, even commercially, with proper attribution. For these reasons, we cannot publish previously copyrighted maps or satellite images created using proprietary data, such as Google software (Google Maps, Street View, and Earth). For more information, see our copyright guidelines: http://journals.plos.org/plosone/s/licenses-and-copyright.

We require you to either (a) present written permission from the copyright holder to publish these figures specifically under the CC BY 4.0 license, or (b) remove the figures from your submission:

a. You may seek permission from the original copyright holder of Figures 1,2 and 4 to publish the content specifically under the CC BY 4.0 license.

We recommend that you contact the original copyright holder with the Content Permission Form (http://journals.plos.org/plosone/s/file?id=7c09/content-permission-form.pdf) and the following text:

“I request permission for the open-access journal PLOS ONE to publish XXX under the Creative Commons Attribution License (CCAL) CC BY 4.0 (http://creativecommons.org/licenses/by/4.0/). Please be aware that this license allows unrestricted use and distribution, even commercially, by third parties. Please reply and provide explicit written permission to publish XXX under a CC BY license and complete the attached form.”

Please upload the completed Content Permission Form or other proof of granted permissions as an "Other" file with your submission.

In the figure caption of the copyrighted figure, please include the following text: “Reprinted from [ref] under a CC BY license, with permission from [name of publisher], original copyright [original copyright year].”

b. If you are unable to obtain permission from the original copyright holder to publish these figures under the CC BY 4.0 license or if the copyright holder’s requirements are incompatible with the CC BY 4.0 license, please either i) remove the figure or ii) supply a replacement figure that complies with the CC BY 4.0 license. Please check copyright information on all replacement figures and update the figure caption with source information. If applicable, please specify in the figure caption text when a figure is similar but not identical to the original image and is therefore for illustrative purposes only.

The following resources for replacing copyrighted map figures may be helpful:

USGS National Map Viewer (public domain): http://viewer.nationalmap.gov/viewer/

The Gateway to Astronaut Photography of Earth (public domain): http://eol.jsc.nasa.gov/sseop/clickmap/

Maps at the CIA (public domain): https://www.cia.gov/library/publications/the-world-factbook/index.html and https://www.cia.gov/library/publications/cia-maps-publications/index.html

NASA Earth Observatory (public domain): http://earthobservatory.nasa.gov/

Landsat: http://landsat.visibleearth.nasa.gov/

USGS EROS (Earth Resources Observatory and Science (EROS) Center) (public domain): http://eros.usgs.gov/#

Natural Earth (public domain): <http://www.naturalearthdata.com/>

We are compliant with the CC BY 4.0 license terms and added the following to the applicable figure captions:

Figure 1: “*The figure contains public domain data from Natural Earth (*[*http://www.naturalearthdata.com/*](http://www.naturalearthdata.com/)*) and public domain location data from the International Tree Ring Databank (*[*https://www.ncei.noaa.gov/products/paleoclimatology/tree-ring*](https://www.ncei.noaa.gov/products/paleoclimatology/tree-ring)*)*”

Figure 2: “*The figure contains public domain data from Natural Earth (*[*http://www.naturalearthdata.com/*](http://www.naturalearthdata.com/)*) and an original spatial layer developed from open-access EVI2 data (*[*https://lpdaac.usgs.gov/products/mcd12q2v006/*](https://lpdaac.usgs.gov/products/mcd12q2v006/)*)*”

Figure 4: “*The figure contains public domain data from Natural Earth (*[*http://www.naturalearthdata.com/*](http://www.naturalearthdata.com/)*) and original results generated in this study.*”

**Additional Editor Comments:**

The reviewers point out technical shortcomings, particularly in the selection of precipitation datasets and in the statistical analysis on the significance on difference in their association with vegetation data and their robustness to, for example, the choice of metric (e.g. R2 vs. rank correlation). These should be addressed in a revision.

We appreciate the reviewer’s comments, and included another high-quality remote-sensing based product to better reflect the state of the art remote-sensing based precipitation estimates. We are glad that we expanded our analysis, and the recommended dataset (MSWEP) performed very well, with improvements especially pronounced where weather station coverage is generally low (e.g. Africa). This was in fact our original working hypothesis, and what we were hoping to demonstrate with this study. So, we are grateful to the reviewers for pointing us in the right direction. Regarding other technical suggestions, please see our replies to each of the reviewer’s comments below.

**Reviewer’ comments to questions:**

1. Is the manuscript technically sound, and do the data support the conclusions?

The manuscript must describe a technically sound piece of scientific research with data that supports the conclusions. Experiments must have been conducted rigorously, with appropriate controls, replication, and sample sizes. The conclusions must be drawn appropriately based on the data presented.

Reviewer #1: Partly

Reviewer #2: Partly

2. Has the statistical analysis been performed appropriately and rigorously?

Reviewer #1: Yes

Reviewer #2: Yes

3. Have the authors made all data underlying the findings in their manuscript fully available?

The PLOS Data policy requires authors to make all data underlying the findings described in their manuscript fully available without restriction, with rare exception (please refer to the Data Availability Statement in the manuscript PDF file). The data should be provided as part of the manuscript or its supporting information, or deposited to a public repository. For example, in addition to summary statistics, the data points behind means, medians and variance measures should be available. If there are restrictions on publicly sharing data—e.g. participant privacy or use of data from a third party—those must be specified.

Reviewer #1: Yes

Reviewer #2: No

We note that all data we use in this study are already open access. That said, we have now deposited our code and intermediate data products (e.g. extracted and summarized remote sensing data) in an open access repository. The link has been included in the manuscript and in the cover letter: <https://doi.org/10.6084/m9.figshare.24540928>

4. Is the manuscript presented in an intelligible fashion and written in standard English?

PLOS ONE does not copyedit accepted manuscripts, so the language in submitted articles must be clear, correct, and unambiguous. Any typographical or grammatical errors should be corrected at revision, so please note any specific errors here.

Reviewer #1: Yes

Reviewer #2: Yes

5. Review Comments to the Author

**Reviewer #1 comments:**

This manuscript evaluates a marginally novel idea (i.e., the use of EVI and tree rings to validate global precipitation datasets). But in the end there's just not much new here. For example, the results show that the products agree in areas with good data coverage, which has been well established in the interpolation literature for a long time now. The most notable result otherwise is that UDEL-TS performs better prior to the 1930s (mostly in North Asia and the Himalayas) according to the tree-ring benchmark, which is a feel-good moment for the Blue Hens out there but not really a major finding for climate science in general. Finally, there's no particular rationale for the satellite and reanalysis products used in the study; prominent satellite product omissions include IMERGEHHE, CHIRPS, GPCP, and MSWEP, and important reanalysis omissions include MERRA-2, CFSRv2, and JRA-55. Some of these omissions are known to outperform some of the products included in the manuscript.

We concede the point that PERSIANN does not include the state of the art with regards to remote sensing based precipitation estimation. However, the included re-analysis products CHELSA and ERA5 are recent, well-regarded and widely used. While we can’t include everything, we added a product from your list that combines gauge, re-analysis and satellite data, available at high resolution (MSWEP). MSWEP did indeed perform very well, with improvements especially pronounced where weather station coverage is generally sparse (e.g. Africa). This was in fact our original working hypothesis, and what we were hoping to demonstrate with this study. So, we are grateful that you pointed us in the right direction.

**Reviewer #2 comments:**

Review of ‘Validation of global precipitation time series products against tree ring records and

remotely sensed vegetation greenness’ by Manvailer and Hamann

This paper compares six different global gridded precipitation products. Performance is evaluated by examining yearly correlations (variance explained) against two biological indices - vegetation greenness and tree ring data. I believe that this approach is novel and warrants publication.

Thank you for your assessment. Please see our replies below for how we have addressed your concerns with additional analysis or revising the manuscript text.

However, there are aspects of the paper that I believe need to be addressed prior to publication. The main issues are:

-I need to be convinced about the validity of many conclusions drawn by the authors. For example, the authors state that: 'The results show that for areas with high density of weather station coverage (e.g. the United States and Europe), all products provide near-identical estimates.' This statement is misleading since all products provide near-identical estimates for all zones, with no particular distinction for Europe and North America. Given the minimal differences (with the exception of PERSIANN), there is a need for statistical testing to determine if any of the differences are actually statistically significant. A Kolmogorov-Smirnov (KS) distribution test prior to averaging the data could be an option, but I'm sure there are others. Ultimately, one could argue that the main conclusions of this study should be: 'All products behave essentially the same over most of the globe, with the exception of PERSIANN.'

Regading our statement '*The results show that for areas with high density of weather station coverage (e.g. the United States and Europe), all products provide near-identical estimates*' you are absolutely right! Although the statement is true, we don’t actually show it in this study.

Our focus is on using biological data for an independent validation of one aspect of climate data quality: how well climate variability tracks temporal variation in biological records. As noted in the introduction, “*this is only a partial validation, because the accuracy of absolute climate values cannot be assessed with biological data sources*.”

So, while we are familiar with the data and we know that the absolute values of estimates are very close in regions of high station density in Europe and the US (whereas they are not elsewhere), this sentence should not appear in this paper, especially not in the abstract. We deleted it. Instead we focus on the correlations with biological data (tree ring and EVI time series). Please see what edits we made in response to your other comments below. Thank you for highlighting this.

We are not so sure about following the recommendation for statistical testing. Statistical testing must make an inference from a sample to a population, and ideally contribute to testing a scientific hypothesis (i.e., testing an explanation for a scientific hypothesis). We are reluctant to contribute to reflexive statistical testing of any difference one may come across.

In our case, because of the gigantic sample sizes that arise from remote sensing data, even minute differences will likely be highly significant, statistically, but that does not have any practical meaning. Furthermore, climate data is highly spatially autocorrelated so we would violate the important assumption of independence (that’s why the p-values will be so highly significant). Then, lastly we would argue that we actually work with the population already (global remote sensing coverage). Statistical tests are moot in this case, because whatever difference we observe, that’s the difference. It needs to be described and it’s not subject to testing of a null-hypothesis. We apriori know that the null-hypothesis is false.

The situation is different for tree ring data, which is a true sample (thousands of trees), and we could draw inferences on the population (all trees in the world). Statistical testing could be useful here, but we are actually not drawing inferences on forest but on global climate data sets. We are again stumped by spatially autocorrelations, which will inflate our p-values to high significance without any meaning. We don’t think we have an application for statistical testing. We simply describe the variance explained in climate data products by available biological data. This is completely sound reasoning and we argue that there is no good opportunity to use statistical testing to make a valid inference.

-The claim that UDEL-TS was superior to all other products for long-term records is somewhat misleading. It would be more accurate to clearly state that it was better than only two other products and was not superior for analyzing long-term time series, but only prior to 1930. When considering the full length of the time series, one could argue that GPCC is the superior product.

OK, we agree. We revised the results and discussion section as follows:

“*Variance explained by moving averages of 21-year windows reveal that the UDEL is generally superior for early climate estimates based on data from 1901-1940, corresponding to R^2^ values of moving windows from 1910-1930 (Fig 5, global panel). Subsequently, R^2^ values are fairly similar for moving window mid-points from 1930 to 1960, and then separate by a moderate amount for the most recent decades, with GPCC performing best, followed by UDEL and CRU4. It is important to note that the superior performance of UDEL in the early decades only applies to specific regions, namely Northern Asia and the Himalayas region, where whether station coverage is generally sparse, and we speculate that the researchers developing the UDEL product may have had access to station records not available for interpolation in CRU and GPCC products. … Regional breakdowns suggest that all three gauge-based climate data products perform fairly similarly besides regional differences in the early decades (Fig 5, regional panels). That said, a small but notable difference among data products is CRU outperforming other data products for recent climate since the 1970s for South America. For the Oceania region GPCC appears to be the best choice for most of the evaluated time series.*”

-The paper does not discuss or mention the scales of the different datasets and the potential impact this might have on the results. Tree ring data, for example, is a very local product with a lot of spatial variability.

Very good. We should absolutely report our sample sizes for regional plots in Figure 5. Our inferences are absolutely not equally reliable for all regions. This is now transparent by including the number of chronologies available for each regional summary.

-The limitations of the study could be much better outlined. There are many possible measures of fitness when comparing products, and the one used in this study (correlation) is useful but gives a rather incomplete view. A highly correlated product could still have a large bias and high RMSE, thereby strongly limiting its usefulness. The authors acknowledge that they cannot evaluate bias, but do so without any further discussion. In my field, a product with a large bias would be automatically discarded. Finally, while monthly average values are useful, daily data is also critical for many applications, making sub-monthly variability important.

Yes, our evaluation does have a specific target audience, but that encompasses a rather sizable group of researchers. I find this may provide directions to anyone who analyzes historical time series in biological or geophysical data. For establishing a putatively causal link between climate and biological response, bias is actually not a big issue. Data can actually be extremely biased without causing problems, e.g., where coarse resolution gridded data is used in mountainous terrain. Datasets like UDEL or CRU have 0.5° resolution, so absolute climate values for temperature variables that strongly vary along elevation gradients are completely off for most samples in a ~50x50 km grid cell. Yet, in the field of dendroclimatology this poses no problem, because a warmer than average summer in a particular year is warmer than average at all nearby positions in a valley position, mid slope, and at high elevation. All correlative/associative analysis approaches will still work perfectly well.

We do agree that we need to highlight that in our evaluation showing that this is relevant for this type of analysis and not for other research that requires unbiased estimates. For example, analyses that focus on climatic comparisons among different locations for a fixed time period, for example ecological niche modeling or species distribution modeling. We added the following to the discussion under the sub-title “Limitations of this analysis”:

“*As noted previously, our contribution is only a partial validation of climate data products, because the accuracy of absolute climate values cannot be assessed with biological data sources. Our evaluation is therefore only relevant for a specific target audience of researchers who analyze historical time series in biological or geophysical data. To establish a putatively causal link between climate and biological response, with correlative or associative methods using time series data, bias is not an important metric. Climate data can actually be extremely biased without compromising such analyses. To give an example, consider the usage of coarse resolution gridded temperature data used in mountainous terrain. Widely used datasets for this purpose such as CRU or UDEL have 0.5° resolution, and absolute temperature values along elevation gradients can vary extremely across an approximately 50×50 km grid cell in mountainous terrain. Yet, in the field of dendroclimatology this poses no problem, because a warmer than average growing season in a particular year is warmer than average at all elevations. That said, our evaluation should not inform data selection for types of analysis that require unbiased estimates. To give an example, analysis types that build on climatic comparisons among different locations for a fixed time period, such as ecological niche modeling or species distribution modeling should not rely on climate data recommendations from this analysis.*”

I am not sure why the authors were surprised by the PERSIANN results. There are many studies that have shown (either by comparison against stations, or using hydrological models) that PERSIANN is not as good as most other available products.

We concede the point that PERSIANN does not include the state of the art with regards to remote sensing based precipitation estimation. However, the included re-analysis products CHELSA and ERA5 are recent, well-regarded and widely used. While we can’t include everything, we added a product suggested by Reviewer #1 that combines gauge, re-analysis and satellite data, available at high resolution (MSWEP). MSWEP did indeed perform very well, with improvements especially pronounced where weather station coverage is generally sparse (e.g., Africa). This was in fact our original working hypothesis, and what we were hoping to demonstrate with this study. So, we are grateful that both reviewers pointed us in the right direction.

L10 ‘are’ instead of ‘is’

Fixed, thank you.

L42 challenging and not challenging

Corrected.

L101 Why were those 6 products chosen and not any others. For example, Beck et al, 2017 did a comparison of 22 precipitation datasets. Beck, H. E., Vergopolan, N., Pan, M., Levizzani, V., Van Dijk, A. I., Weedon, G. P., ... & Wood, E. F. (2017). Global-scale evaluation of 22 precipitation datasets using gauge observations and hydrological modeling. Hydrology and Earth System Sciences, 21(12), 6201-6217.

Yes, it’s always possible to do more, but we selected widely used and widely cited global datasets. Our primary goal as stated in the objectives section was to see if different types of data (re-analysis and remote-sensing based) can provide better estimates in regions with poor weather station coverage. As noted before, we concede the point that we did not pick a well-regarded satellite-based product, but we have now included a high-quality satellite-based product (multi-source, in fact) that does perform very nicely. This did change our conclusions and we are grateful to for both reviewers to recommend inclusion of a high-quality satellite based product.

L!35 I looked very hard and could not find Table 1. I believe it has not been submitted.

Now included directly in the manuscript.

L151-152 why was long and medium frequency filtered out? These are important characteristics of precipitation.

This step must be applied to tree ring data to remove age-related trends. We strictly evaluate tree-ring response to interannual climate variability. It is standard procedure to remove long-term trends in tree ring data as any long term trends in climate data would be confounded with age effects. There are really no alternatives to this step, unless an age-cohort sampling scheme is designed for trend analysis. We added the following explanation to this section:

“*Chronologies were detrended using the Friedman Super-Smoother method to remove both long- and medium-frequency variability and maintain short-frequency (year-to-year) variability. This removes age-related trends in tree ring data that may be confounded with long-term climate trends or decadal climate oscillations. Our analysis therefore evaluates how well tree ring data tracks inter-annual climate variability.*”

L194 not clear what is meant by ‘dark gray coverage’. And I really studied the Figure.

Sorry, this was not shown in Fig. 3, but in Fig. 2. Thank you for noticing this mistake. We are very grateful for your very close reading that seems to turn up all of our mistakes (hopefully not too many).

L204-205 Not entirely clear as to what was used for the variance estimation. What is the cumulative annual EVI area under the curve ? If I get this right, a single value at 8 months is compared to the cumulative precipitation for the first 8 months for each year ? Please clarify.

Yes, it’s a single annual EVI area under the curve value evaluated against the 8 months of precipitation data that are most likely to influence that EVI value. Any monthly climate data past the end of the growing season can obviously no longer have an influence, so our last month included in the northern hemisphere was August, and the last month for the southern hemisphere was February. So, for the southern hemisphere the monthly data evaluated would be January and February of the current year, and July to December of the previous year to explain the growing-season area under the curve EVI value.

Conclusion is essentially a copy of parts of the abstract, I believe the space could be used much more efficiently.

Yes, we definitely don’t want the conclusions to repeat the abstract. Rather, the section should expand on just the last sentences of the abstract (important findings, take-home messages, synthesis). The inclusion of MSWEP required that we changed both the abstract and conclusions. The revised abstract section read as follows:

“*The results for the 20-year EVI based validation shows that all gauge and re-analysis data products performed similarly, but were outperformed by the multi-source MSWEP product, especially in regions with low weather station coverage, such as Africa. For analyzing long 120-year time-series, UDEL-TS showed superior performance prior to the 1940s, with especially large margins for northern Asia and the Himalayas region. For other regions, CRU-TS and GPCC could be recommended. We provide maps that can guide the best regional choice of climate product for research involving time series of biological response to historic climate variability and climate change.*”

In the conclusion section, we now expand on this as follows:

“*For researchers who analyze historical time series in biological or geophysical data our analysis allows to infer the best regional choice of time-series precipitation products. That said we should emphasize that for applications that require unbiased estimate of differences among locations for a fixed time period, our recommendations are not applicable. The results for the 20-year time series of remotely sensed EVI allows for comprehensive regional comparisons. All gauge and re-analysis based data products performed similarly, but they were outperformed by the multi-source MSWEP product that utilizes gauge, re-analysis, and remote sensing data. Especially for regions with low weather station coverage, such as Africa, the margin of improvement in how well MSWEP time series data tracks vegetation greenness was impressive. The analysis confirmed our working hypothesis that satellite-based or multi-source products have the potential to provide better precipitation estimates in regions of low weather station coverage. Unfortunately, satellite or re-analysis based products are not available prior to the 1980s. For applications that require longer time series analysis, gauge based products are the only option. Of the three gauge-based data products included in the analysis against 120-year tree ring records (CRU, GPCC and UDEL), the results showed that UDEL had superior performance prior to the 1940s, with especially large margins for northern Asia and the Himalayas region. Form the 1940s onward, all three gauge-based climate data products perform fairly similarly. Locally, CRU outperformed other data products after the 1960s for South America. For the Oceania region GPCC was the best choice for analysis of long time series.*”
